# Photo-responsive nanoporous liquid crystal polymer films for selective dye adsorption†

**DOI:** 10.1039/d3ra06791b

**Published:** 2024-01-02

**Authors:** Hongju Zeng, Yun Wang, Changxiang Li, Junjie Ren, Runzi Lu, Huiyao Zhang, Xi Wang, Xingbin Lv, Hairong Yu, Ting Liang, Changjing Cheng

**Affiliations:** a College of Chemistry and Environment, Southwest Minzu University Chengdu Sichuan 610200 PR China liangting@swun.edu.cn chengcj@swun.edu.cn; b Key Laboratory of Fundamental Chemistry of the State Ethnic Commission Chengdu Sichuan 610200 PR China; c Chengdu BOE Display Sci-tech Co. Ltd. Chengdu Sichuan 610200 PR China

## Abstract

Photo-responsive nanoporous polymer films (AZOF-R(NC6)) have been developed by a template method based on a hydrogen-bonding supramolecular liquid crystal (LC) and a light-sensitive azobenzene LC crosslinker in this work. Anionic nanopores were obtained after the removal of template NC6 using KOH solution. The AZOF-R(NC6) demonstrates charge-selective dye adsorption and the maximum adsorption capacity for Rh6G is 504.6 mg g^−1^. The AZOF-R(NC6) film without UV light treatment shows a 32% higher adsorption capacity for Rh6G than the AZOF-R(NC6) film treated with UV light within the initial 10 min. In addition, UV light can trigger the release of the adsorbed dye from the polymer film due to the pore size change arising from the *trans*–*cis* isomerization. Besides, the used polymer film can be effectively regenerated using a HCl solution. Such functional polymer films with highly ordered nanopores and photo-responsive properties hold great promise in selective adsorption and mass separations.

## Abstract 2

A photo-responsive nanoporous polymer film (AZOF-R(NC6)) has been fabricated. AZOF-R(NC6) shows charge-selective and light-responsive dye adsorption.

## Graphical abstract

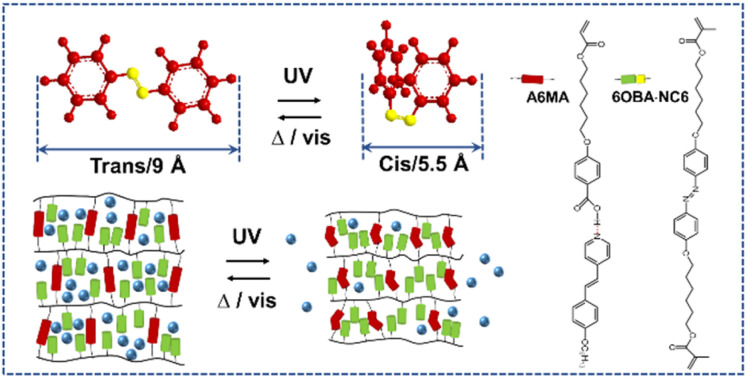

## 1. Introduction

Nanoporous materials with controllable pore sizes and surface properties are of interest in multiple fields including mass separations, drug delivery, catalysts, batteries and sensors.^1–6^ For those significant applications, two key challenges should be addressed in advance. The first one is to achieve nanoporous materials with tailored pore sizes and programmed surface properties. The host–guest template method based on self-assembly of non-covalent bonds such as hydrogen bonds is an effective strategy to create nanoporous materials with desired pore sizes.^7,8^ The templates can be easily removed after solidifying the structures and the obtained pores can reproduce the properties of the templates, thus yielding nanosized pores. Another one is to realize stimuli-responsive capture, transport, or release of cargoes from those nanopores in the materials.^9,10^ Various external stimuli including temperature, light, magnetic and electric fields, pH and ions have been utilized to realize those functions. Light is an attractive stimulus due to its accurate and remote control.^11^ Azobenzene is a widely studied photosensitive compound,^12,13^ which can undergo *trans*–*cis* isomerization upon light irradiation, thus producing changes in size, conformation, and dipole moment.^14–16^ Integrating a template method with photosensitive azobenzene to fabricate light-responsive nanoporous materials is a feasible pathway to realize those significant applications such as selective adsorption and separations.

Polymer liquid crystals (LCs) are a kind of functional material with highly ordered structures, which is greatly beneficial to form nanopores with desired pore properties.^17–19^ Hydrogen-bonding supramolecular LCs integrated with a template method have attracted widespread attention for the past few decades due to their modifiable and reversible hydrogen-bonding behaviors.^20–23^ Polymerization of hydrogen-bonding monomers followed by templates removal can produce functional polymer films with well-organized nanopores and oriented shapes. This extremely benefits their actual applications in selective adsorption and separations.^20–23^ The Gin^24–26^ and Kato^27–30^ groups have recently developed various functional polymer films using cubic phase LCs to realize the selective adsorption and separations for glucose, bacteria, and various ions (such as Br^−^ and SO_4_^2−^). Feng *et al.*^31^ developed a nano-filtration film by a template method based on lyotropic LCs for selective dye adsorption and separation. Introducing light-sensitive azobenzene groups in LCs provides a simple and effective approach to realize the light-controlled functions. Kuringen *et al.*^32^ constructed photo-responsive nanoporous polymer films by using azobenzene crosslinker in the fabrication of smectic LC hydrogen-bonding polymer networks; the number of binding sites and pore size changed under UV irradiation due to the *trans*–*cis* isomerization of azobenzene groups. Although some light-responsive nanoporous films have been developed^33^ the photo-controlled capture and delivery behaviors of species from those nanoporous materials are rarely reported.

**Fig. 1:**
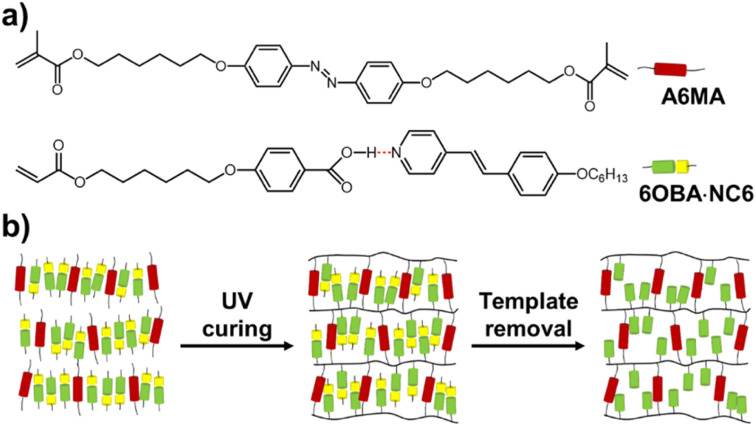
(a) Chemical structures of the monomer mixture containing the hydrogen-bonding 6OBA·NC6 complex and the azobenzene crosslinker A6MA. (b) Schematic illustration of the fabrication process of the nanoporous polymer films.

Recently, we developed a two-dimensional (2D) nanoporous smectic polymer film based on the hydrogen-bonding supramolecular LC assembly *via* a template method and the obtained nanoporous functional film displays anisotropic, size- and charge-selective dye adsorption.^34^ To further demonstrate the light-controlled dye adsorption capability of the film, we fabricated a photo-responsive nanoporous LC polymer film (AZOF-R(NC6)) by using a light-sensitive azobenzene crosslinker in the hydrogen-bonding smectic LC polymer network in this work. The NC6 small molecules (Fig. 1a) serve as hydrogen-bonding acceptors to trigger LC phases as well as templates to create anionic nanopores. The azobenzene crosslinker realizes the light-responsive dye-adsorption function of the film. Our fabricated AZOF-R(NC6) shows charge-selective dye adsorption thanks to the anionic nanopores with rich negative charges after KOH treatment. The adsorption of cationic dye Rh6G follows the quasi-second-order kinetic model. Upon UV light irradiation, the smectic layer spacing reduces arising from the pore size change. The absorption rate of Rh6G on the AZOF-R(NC6) is slower than that without UV light irradiation. Besides, the Rh6G-loaded AZOF-R film can be easily regenerated using a HCl solution. Such functional polymer films with highly ordered nanopores and light-responsive property hold great promise in selective adsorption and mass separations.

## 2. Experimental section

### 2.1. Materials

The LC azobenzene crosslinker 4,4′-bis(6-methacryloyloxy hexyloxy)azobenzene (A6MA, Fig. 1a) were synthesized in our laboratory. The hydrogen-bonding donor 4-(6-acryloxy-hexyl-1-oxy)-benzoic acid (6OBA, Fig. 1a) was purchased from Meryer Chemicals (Shanghai, China). The hydrogen-bonding acceptor (4-(4′-hexyloxy)styryl)-pyridine (NC6). The photoinitiator 1-hydroxycyclohexyl phenyl ketone (Irgacure 819) and thermal initiator *p*-methoxyphenol were obtained from Sigma-Aldrich. Dyes including methylene blue (MB), methyl orange (MO) and rhodamine 6G (Rh6G) were bought from Jinshan chemicals (Chengdu, China). The alignment agents polyimide ZKPI-4220 (planar) was obtained from POME Technology Co., Ltd. (Beijing, China). All other chemicals were of analytical grade and were used as received. Deionized water (18.25 MΩ, 25 °C) from a Milli-Q plus purification system (Millipore) was used throughout the experiments.

### 2.2. Preparation of the LC polymer films

The azobenzene LC polymer film was prepared from a mixture of 6OBA·NC6 (90 wt%) and azobenzene crosslinker A6MA (10 wt%). In preparing the supramolecular complex, 6OBA and NC6 are used in a 1 : 1 molar ratio, which aligns with the stoichiometry necessary for optimal hydrogen bonding. Irgacure 819 (0.5 wt%) was added to initiate the polymerization. *p*-Methoxyphenol (0.1 wt%) was added to prevent the thermal polymerization. All compounds were dissolved in dichloromethane to form a homogeneous solution and the dichloromethane was then removed by natural evaporation. The monomer mixture was dried in vacuum before use. Azobenzene LC polymer film was obtained through photopolymerization reaction in a home-made glass cell and the thickness of the film (5 μm) was determined by the cell gap. The fabrication process of the nanoporous polymer films was schematically shown in Fig. 1b. The monomer mixture was filled into the cell by capillary suction in the isotropic phase (140 °C), and then cooled down to the liquid crystal phase (90 °C) for the subsequent reaction. To prevent the *trans*–*cis* isomerization of azobenzene groups, the reaction was conducted using a UV light equipped with 400 nm cut-off optical filter for 1 h. Then the film was thermally cured at 120 °C for another 20 min after UV curing to guarantee the complete reaction. To obtain the functional film with planar alignments, the glass slides (LC cell) needed to be treated with rubbed polyimide (ZKPI-4220).

### 2.3. Preparation of the NC6-removed films

The NC6 template was removed by immersing the film in a KOH solution. The rationale for employing KOH to extract NC6 from the polymer film is based on the pH sensitivity of the hydrogen bonds present within the NC6 complex. These hydrogen bonds are particularly susceptible to cleavage in basic conditions, effectively breaking at a pH above 8.^35,36^ KOH provides a strong alkaline environment which facilitates the disruption of these hydrogen bonds, allowing for the selective removal of NC6. In detail, the pristine films were immersed in a KOH solution of 0.05 M for 30 min under continuous shaking and then washed with ethanol to remove the residual KOH, and finally dried in vacuum prior to the further use.

### 2.4. Characterization

The hydrogen bonds were characterized using a Fourier transform infrared (FT-IR) spectrometer (IR 200, Thermo Nicolet, USA). Polarized optical microscopy (POM) images were obtained using a Caikon microscope (XPF-500C) equipped with polarization filters. A CK-400 hot stage was used to conduct the thermo-controlled experiments. Differential scanning calorimetry (DSC) was performed using a TA instruments Q2000 calorimeter. The samples were heated or cooled at a rate of 10 °C min^−1^ with an isothermal equilibration of 2 min after each heating or cooling ramp. The thermogravimetric analyses (TGA) of samples were performed using a STA-449C (Germany). The samples were heated from 30 to 800 °C at a rate of 10 °C min^−1^ under a nitrogen atmosphere. The XRD patterns were collected on an Anton Paar Saxsess mc2 apparatus attached to an ID 3003 laboratory X-ray generator (General Electric) equipped with a sealed X-ray tube (PANalytical, *λ* (Cu-K_α_) = 0.1542 nm, 40 kV, 50 mA). Adsorption kinetics were studied by UV-vis spectroscopy using a UV-vis spectrometer (Spectrum lab 752s, Shanghai). Other UV-vis spectroscopy experiments were performed using a UV-vis spectrophotometer (UV-1800PC, Mapada Instruments, Shanghai).

### 2.5. Dye adsorption

The dye adsorption performances of the functional LC polymer films were investigated through batch adsorption experiments. Effects of the UV irradiation and pore charges on the dye adsorption were determined. In a typical run, certain mass of functional polymer films was added in dye solutions with predetermined concentrations, and then the samples were sealed and placed in a thermostatic shaking bath under 80 rpm and at 25 °C with preset time interval. Absorbance of the solutions before and after reacting with the films was measured using a UV-vis spectrophotometer in the wavelength range of 300–800 nm. All the adsorption experiments were carried out at least thrice to ensure the accuracy of data analysis. The concentrations of dyes in water before and after adsorption was calculated from the UV-vis data. The adsorption capacity (*q*_e_, mg g^−1^) was calculated by the following equation:1
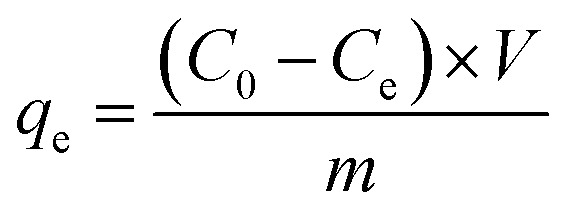
where *C*_0_ and *C*_e_ (mg L^−1^) are the initial and equilibrium concentrations of Rh6G. *V* (L) is the volume of dye solutions, and *m* (g) is the weight of dried films. The equilibrium concentrations of dyes were measured after adsorption for 24 h at 25 °C, and the adsorption kinetics experiments were conducted in the time range of 0–250 min. Aqueous samples including the initial solution sample as the zero point were collected and examined at designated time intervals at 25 °C.

## 3. Results and discussion

### 3.1. Preparation and characterization of the monomer mixture

**Fig. 2:**
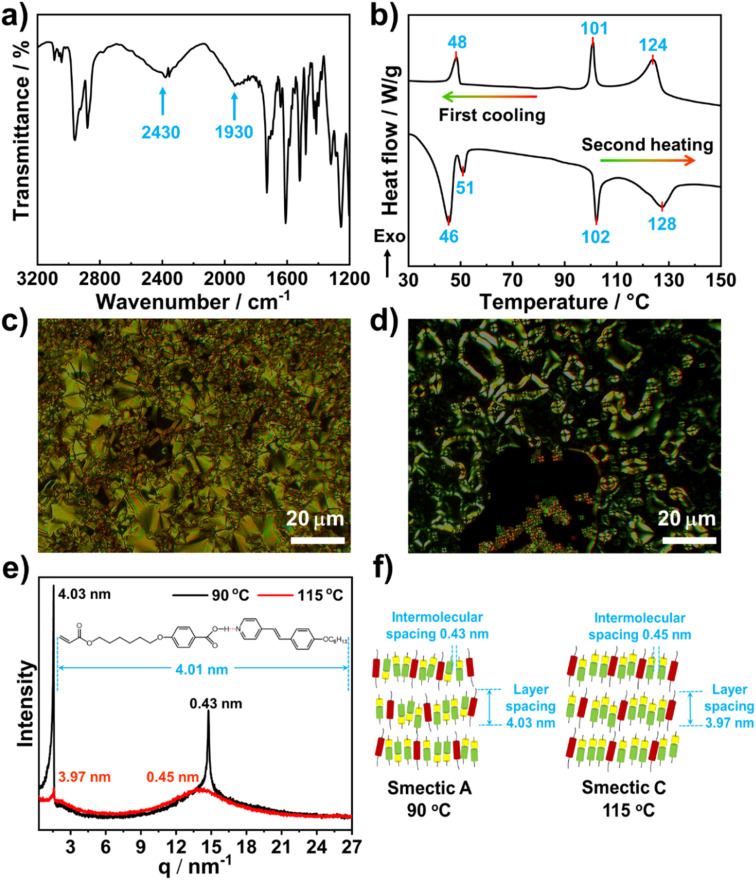
Characterization of the structure and the mesomorphism of the monomer mixture 6OBA·NC6·A6MA. (a) FT-IR spectrum, (b) DSC spectra, (c and d) POM pictures at (c) 90 and (d) 115 °C, (e) XRD spectra and (f) schematic diagram of the 6OBA·NC6·A6MA at 90 and 115 °C.

The hydrogen-bonding supramolecular monomer mixture (6OBA·NC6) and the azobenzene crosslinker A6MA were studied by FT-IR spectroscopy. As observed from Fig. 2a, two typical peaks at 1930 and 2430 cm^−1^ due to the O–H stretching band and its Fermi resonance reveal the formation of hydrogen bonds between the 6OBA and the NC6 (ref. 37) The DSC results show that the monomer mixture exhibits LC transitions during the heating and cooling processes (Fig. 2b). The mesophases are identified by slow cooling from the isotropic phase using POM observation, which forms a smectic C (SmC) phase from 124 to 101 °C. Upon further cooling, a smectic A (SmA) phase is observed as the temperatures range from 101 to 48 °C, and then the crystallization happens at 48 °C. Fig. 2c and d present the POM pictures of the monomer mixture at 115 and 90 °C during the first cooling process, respectively. The temperature-dependent XRD results of the monomer mixture confirm the presence of the SmC and SmA phases (Fig. 2e). A broad band centered at 14.76 nm^−1^ can be observed from the XRD pattern at 90 °C probably arising from the intermolecular spacing (0.43 nm) in the SmA phase. A sharp peak centered at 1.56 nm^−1^ indicates a layer spacing of 4.03 nm, which is almost the same as the molecular length of the 6OBA·NC6 in the stretching conformation (4.01 nm). For the XRD pattern obtained at 115 °C, a sharp peak centered at 1.58 nm^−1^ shows a lamellar structure with a *d*-spacing of 3.97 nm, which is a slightly smaller than the molecular length of the 6OBA·NC6. A broad band centered at 14.76 nm^−1^ results from the intermolecular spacing of 0.45 nm. These results indicate that with increasing the temperature, the molecular arrangement changes from a SmA phase to a SmC phase. The XRD pattern shows that the 6OBA·NC6·A6MA forms a layered structure with almost fully stretched aliphatic chains in the smectic phase. The proposed smectic arrangement of the 6OBA·NC6·A6MA is schematically illustrated in Fig. 2f.

### 3.2. Fabrication and characterization of the pristine and NC6-removed films

**Fig. 3:**
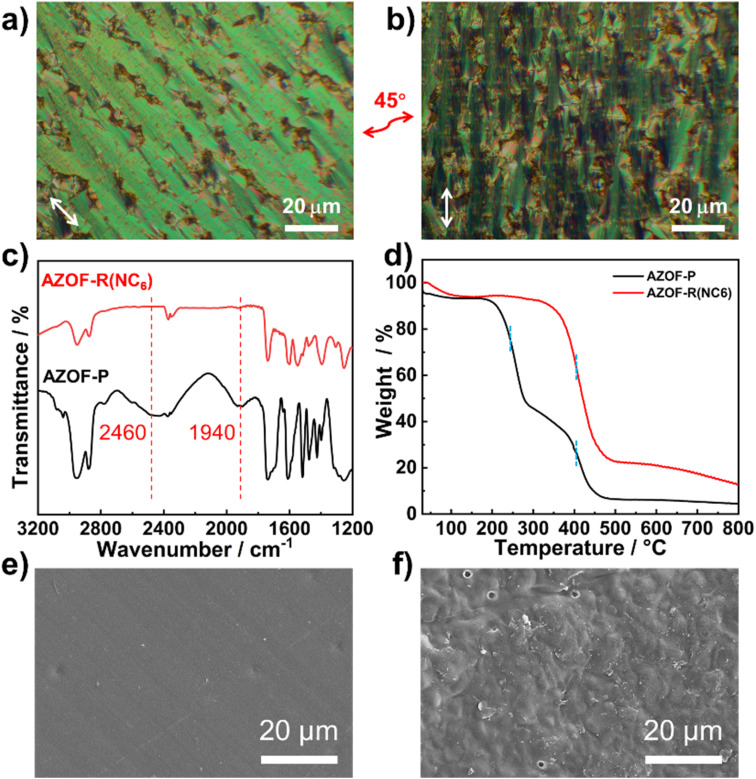
Characterization of the structure and thermal stability of the polymer films. The POM pictures of the (a) planar aligned pristine film and (b) its 45° rotation view. (c) FT-IR spectra and (d) TGA results of the pristine and NC6-removal films. SEM images of the surface of the pristine (e), the NC6-removal (f) films. The arrow represents the direction of alignment.

The polymer film with 5 μm thickness was fabricated by UV-initiated photopolymerization of the monomer 6OBA·NC6·A6MA. The rubbed polyimide ZKPI-4220 glass surface was used to produce the uniform planar alignment. After UV exposure, polymerization of 6OBA·NC6·A6MA is proved by the disappearance of C=C stretching vibration peak at 1640 cm^−1^ (Fig. S1†). The planar alignment of the film was studied by POM observation. As shown in Fig. 3a and b, obvious bright and dark changes are observable upon rotating 45°, showing a good planar alignment of the film. The nanoporous films were obtained through removing the template NC6 using KOH solution since hydrogen bonds could break under an alkaline condition. The FT-IR result verifies the removal of NC6, as shown in Fig. 3c, the disappearance of the peaks at 1940 and 2475 cm^−1^ indicates the cleavage of the hydrogen bonds, while the characteristic peaks of the carboxylate at 1549 and 1395 cm^−1^ become stronger. This provides a direct evidence that KOH has penetrated the film network, and broken the hydrogen bonds and deprotonated the acid groups (–COOH) into –COO^−^ groups. The thermal stability of the films was investigated by TGA technique. The weight loss of the AZO-P film varies with increasing the temperature and a two-step weight loss is noticeable (Fig. 3d) due to the decomposition of NC6 and the polymer chains, respectively. For the AZO-R(NC6) film, one-step weight loss representing the decomposition of the polymer chains is observed. This result indicates that the film has occurred to polymerize and possesses good thermal stability. Fig. 3e and f show the typical SEM images of the surface of the pristine film and the NC6-removal film, which depict distinct surface characteristics. The pristine film exhibits a smooth surface, contrasting with the rough surface observed in the NC6-removal film, signifying the formation of nanopore structures upon NC6 removal.

### 3.3. Dye adsorption by the functional films

**Fig. 4:**
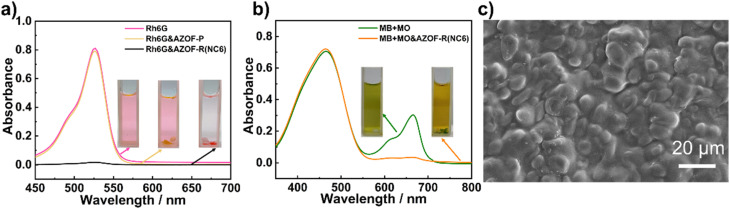
(a) UV-vis spectra and the corresponding samples (the inset) of Rh6G solutions before and after adsorption by the AZO-P and AZO-R(NC6) films for 24 h. (b) Charge-selective MB adsorption over MO. UV-vis spectra and vials (the inset) of the dye solutions before and after adsorption. (c) SEM image of the surface of the dye adsorption film.

To ascertain the significance of the nanoporous structure on the dye adsorption, both the AZO-P and the AZO-R(NC6) films were used to study the dye adsorption. As observed in Fig. 4a, three cuvettes contain pure Rh6G solution, Rh6G solutions reacted with the AZO-P film and the AZO-R(NC6) film, respectively. The Rh6G solution after adsorption using the AZO-R(NC6) film becomes nearly colorless, and the dye-loaded film appears pink, indicating that the AZO-R(NC6) film can strongly adsorb Rh6G molecules. The maximum adsorption capacity of Rh6G is 504.6 mg g^−1^. However, the color of the dye solution after interacting with the AZO-P film changes little, showing worse Rh6G adsorption of the film. Absorbance spectra of the dye solutions after adsorption with the AZO-R(NC6) film keeps nearly straight, indicating a large Rh6G uptake of the film. However, no evident difference in the absorbance spectra is observed for the dye solution adsorbed by the AZO-P film (Fig. 4a), revealing its inability to adsorb the Rh6G. These results indicate that the nanoporous structure in the AZO-R(NC6) film plays an important role in the Rh6G adsorption. This is due to that the anionic nanopores is generated after KOH treatment to remove NC6 template from the film. The presence of abundant carboxylate (–COO^−^) groups endows the film with sufficient charges and high specific surface area, which greatly benefits from the adsorption of cationic Rh6G. Fig. 4c shows the SEM image of the surface of the dye adsorption film. After Rh6G adsorption, surface roughness of the film increased obviously. Furthermore, the surface exhibits swelling state, indicating the occupation of nanopores by Rh6G molecules.

Competitive dye adsorption experiments were performed to investigate the charge-selective adsorption of the AZO-R(NC6). Solutions consisting of anionic MO and cationic MB dyes, both have a similar molecular size, were dissolved in water to obtain a green solution (the inset in Fig. 4b). The UV-vis spectrum of the solution shows two peaks, one at 465 nm corresponding to MO and another at 664 nm ascribable to MB. After interaction for 24 h, a yellow solution and a MB-loaded green AZO-R(NC6) film are observed, implying selective adsorption of MB onto the AZO-R(NC6). The UV-vis spectra of the solution with AZO-R(NC6) film show the same absorbance peak of MO as the initial solution, while the MB peak is invisible (Fig. 4b), strongly indicating the charge selective adsorption of MB onto the film. This is due to that the anionic nanopores are obtained after the removal of NC6 template. Charge interactions play a decisive role in the dye adsorption. Therefore, the AZO-R(NC6) can selectively adsorb the cationic dye MB instead of the anionic dye MO.

Adsorption kinetics is an important factor that affects the adsorption behavior of an adsorbent for adsorbates. Two classical adsorption kinetics models (the Pseudo-first-order and Pseudo-second-order models) were utilized to fit the obtained adsorption data:

Pseudo-first-order model:

2 ln(*q*_e_ − *q*_*t*_) = ln *q*_e_ − *k*_1_*t*

**Fig. 5:**
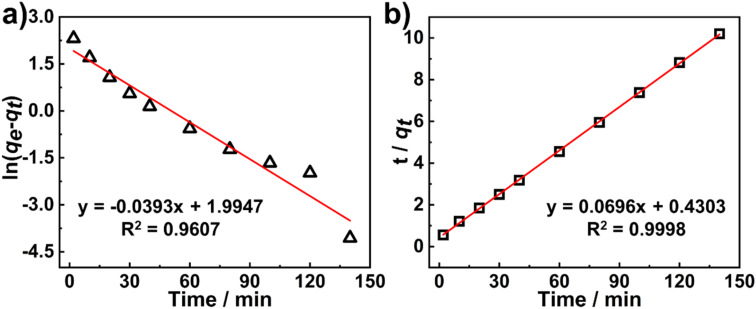
(a) Fitting of the Pseudo-first-order kinetics and (b) the Pseudo-second-order kinetics of Rh6G adsorption onto the AZO-R(NC6) film.

Pseudo-second-order model:3
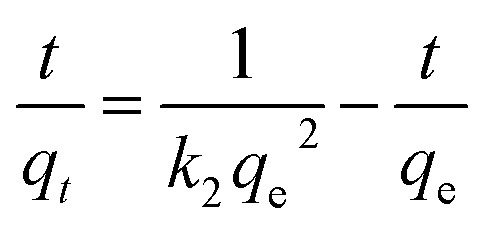
where *q*_e_ and *q*_*t*_ (mg g^−1^) are the adsorption capacities of Rh6G at equilibrium state and at time *t* (min), respectively; *k*_1_ (min^−1^) and *k*_2_ (g (mg min)^−1^) refer to the rate constants of the Pseudo-first-order and Pseudo-second-order models, respectively. The kinetic parameters can be calculated from the slopes and intercepts of the linear plots of ln(*q*_e_ − *q*_*t*_) *versus t* for the Pseudo-first-order model and *t*/*q*_*t*_*versus t* for the Pseudo-second-order model (Fig. 5). All the calculated results were summarized in Table S1†. The results show that the higher *R*_2_ value (0.9998) is obtained for the Pseudo-second-order model, and the calculated adsorption capacity (*q*_e,cal_ = 14.37 mg g^−1^) is closer to the experimental value (*q*_e,exp_ = 13.74 mg g^−1^), indicating that the kinetics of the Rh6G adsorption onto the AZO-R(NC6) film follow the Pseudo-second-order model. The strong electrostatic interactions between the cationic Rh6G and anionic nanopores of the AZO-R(NC6) film contribute to the high adsorption capacity of Rh6G.

**Fig. 6:**
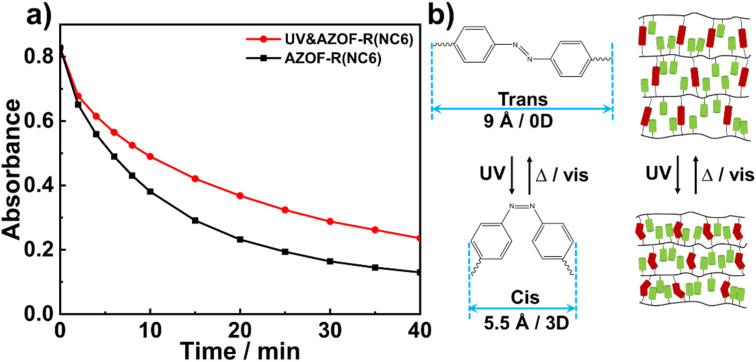
(a) Adsorption kinetics of Rh6G onto the AZO-R(NC6) film and the UV-treated AZO-R(NC6) film. (b) Schematic diagram of the isomerization of the azobenzene groups and the corresponding structures of the AZO-R(NC6).

To study the effect of isomerization of azobenzene groups on the dye adsorption, two AZOF-R(NC6) films with the same quality were prepared under dark condition, one was irradiated using 365 nm UV light for 1 h, while the other one was not suffered from UV irradiation. And both two films were immersed in Rh6G solution (5 mg L^−1^). The Rh6G adsorption at different time was recorded under shaking conditions. As shown in Fig. 6a, the AZOF-R(NC6) film without UV light treatment shows a 32% higher adsorption capacity for Rh6G than the AZOF-R(NC6) film treated with UV light within the initial 10 min. This may be ascribable to the isomerization of azobenzene groups which causes a space change of the pores in the AZOF-R(NC6). As shown in Fig. 6b, the *d*-spacing of the AZOF-R(NC6) is smaller upon UV irradiation, which is caused by the molecular length change of the azobenzene crosslinker under UV exposure.^32^ Thus the diffusion of dye molecules in the film is limited.

### 3.4. Photo-responsive dye release

**Fig. 7:**
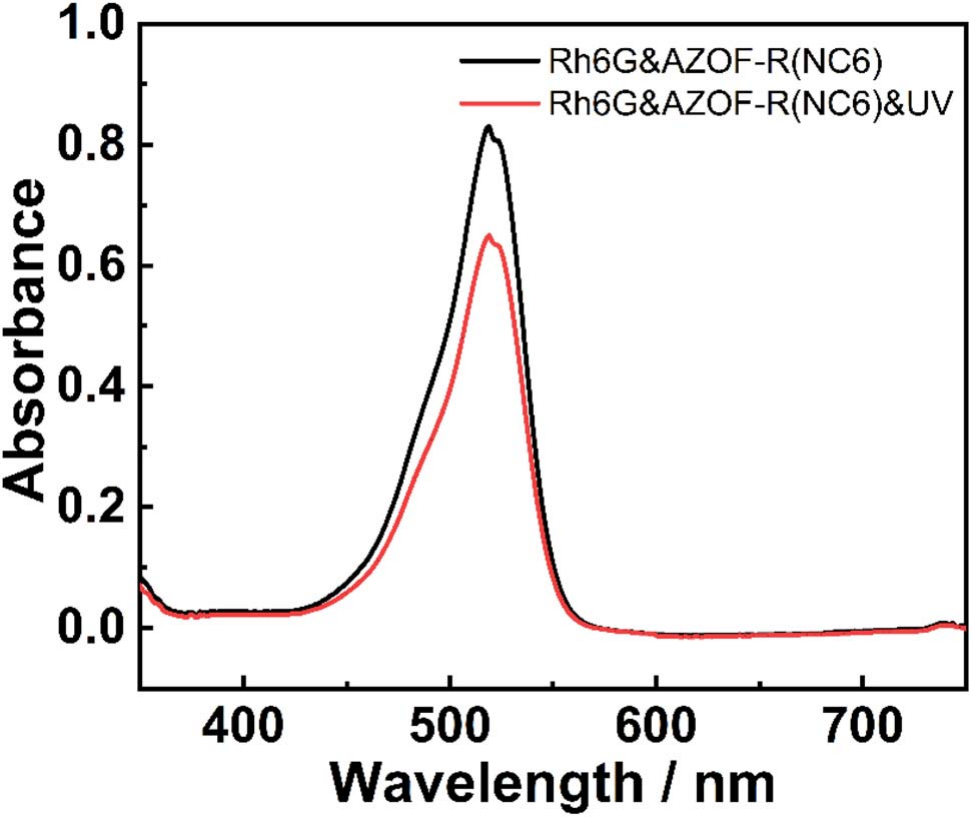
Photo regulated Rh6G release of the AZOF-R(NC6) film.

Inspired by the photo-controlled space change of the film. The photo-responsive release behavior of the AZOF-R(NC6) film for Rh6G was also investigated. The AZOF-R(NC6) film was firstly placed in a 100 mg L^−1^ Rh6G solution under dark conditions to adsorb Rh6G till equilibrium. Then, the film was irradiated with a UV lamp. The change in absorbance before and after irradiation was recorded. As shown in Fig. 7, the absorbance of the solution increased after UV irradiation, indicating that part of Rh6G molecules release into the solution. That is because that the azobenzene groups in AZOF-R(NC6) film undergo a *trans*–*cis* isomerization after UV irradiation, thus resulting in reduction in pore size and delivering part of the adsorbed Rh6G molecules. It should be noticed that the complete release of Rh6G is not realized owing to that the pore size change triggered by the isomerization is limited, and the space squeezing is insufficient to counterbalance the charge interactions between the dye molecules and carboxylic acid groups.

### 3.5. Desorption and reusability

**Fig. 8:**
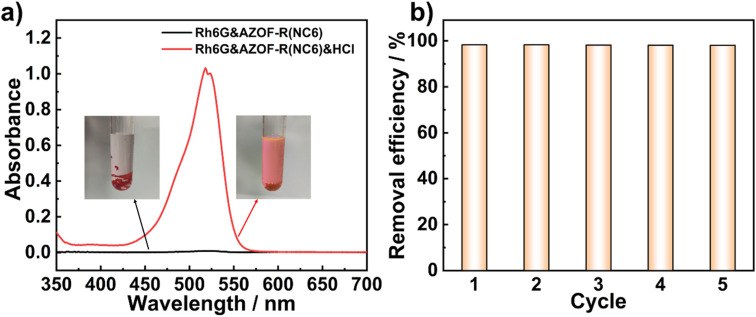
(a) UV-vis spectra and the corresponding solution samples (the inset) before and after regeneration. (b) Reusability of the AZOF-R(NC6) film for Rh6G adsorption.

The regenerability and recyclability of an adsorbent are of importance for its practical applications. Although the photo-controlled dye release is realized, the uptake dyes can not fully release due to a limited structure change of the isomerization. Therefore, a HCl solution was used to regenerate the Rh6G-loaded AZOF-R(NC6) film (*C*_Rh6G_ = 100 mg L^−1^). The COO^−^ groups are protonated to produce –COOH groups under acidic conditions, thus giving rise to the release of the Rh6G molecules from the film. The regenerability is evaluated by the removal efficiency of Rh6G in different cycles. As depicted in Fig. 8a, acid facilitates the release of the dye from the film. Regeneration is achieved through treatment with KOH alcoholic solution for obtaining the anionic nanopores. The AZOF-R(NC6) film exhibits the capacity to adsorb Rh6G from the solution, resulting in a red color of the film and the solution becoming colorless. Subsequent desorption in HCl allows the release of Rh6G from the film, causing the solution to turn red while the film becomes colorless. The FT-IR spectra (Fig. S2†) validate both the adsorption and desorption processes of Rh6G. Specifically, the presence of the –CN stretching vibration peak at 1658 cm^−1^ in Rh6G signifies its adsorption onto the film.^38^ Upon regeneration, this peak disappears, indicating the successful release of Rh6G from the film. UV-vis spectra indicate that the previously adsorbed Rh6G is released efficiently. Over 98% removal efficiency is obtained even after five cycles of adsorption/regeneration, as presented in Fig. 8b, indicating that our polymer LC film owns excellent regeneration performances.

## 4. Conclusions

Novel photo-responsive nanoporous polymer films (AZOF-R(NC6)) have been successfully fabricated by a template method based on the self-assembly of the hydrogen-bonding supramolecular smectic liquid crystals and an azobenzene LC crosslinker. The template NC6 could be effectively removed by KOH treatment and anionic nanopores were achieved. The AZOF-R(NC6) film demonstrates a higher adsorption capacity for cationic dye Rh6G and the maximum adsorption capacity is 504.6 mg g^−1^. Adsorption kinetics is well described by the Pseudo-second-order kinetics model. The AZOF-R(NC6) film without UV light treatment shows a 32% higher adsorption capacity for Rh6G than the AZOF-R(NC6) film treated with UV light within the initial 10 min due to that the pore size of the film changes because of the *trans*–*cis* isomerization of azobenzene groups. In addition, part of the uptake dyes in the polymer film can be released thanks to the pore size shrinkage arising from the *trans*–*cis* isomerization. Besides, our developed polymer LC film possesses an excellent regenerability. Such functional nanoporous films with highly ordered nanopores and light-responsive property as well as excellent regenerability hold great potential in selective adsorption and mass separations.

## Conflicts of interest

There are no conflicts to declare.

## Supplementary Material

RA-014-D3RA06791B-s001

## References

[cit1] Jackson E. A., Hillmyer M. A. (2010). ACS Nano.

[cit2] Sajanlal P. R., Sreeprasad T. S., Samal A. K., Pradeep T. (2011). Nano Rev..

[cit3] Lee J. H., Han K. S., Lee J. S., Lee A. S., Park S. K., Hong S. Y., Lee J. C., Mueller K. T., Hong S. M., Koo C. M. (2016). Adv. Mater..

[cit4] Wu D., Xu F., Sun B., Fu R., He H., Matyjaszewski K. (2012). Chem. Rev..

[cit5] Schenning A. P. H. J., Gonzalez-Lemus Y. C., Shishmanova I. K., Broer D. J. (2011). Liq. Cryst..

[cit6] Adiga S. P., Jin C., Curtiss L. A., Monteiro-Riviere N. A., Narayan R. J. (2009). Wiley Interdiscip. Rev.: Nanomed. Nanobiotechnol..

[cit7] Ishida Y. (2010). Materials.

[cit8] Paleos C. M., Tsiourvas D. (2001). Liq. Cryst..

[cit9] Jochum F. D., Theato P. (2013). Chem. Soc. Rev..

[cit10] Zhai L. (2013). Chem. Soc. Rev..

[cit11] Zhang S., Peng Y., Jiang W., Liu X., Song X., Pan B., Yu H. Q. (2014). Chem. Commun..

[cit12] Natansohn A., Rochon P. (2002). Chem. Rev..

[cit13] El Halabieh R. H., Mermut O., Barrett C. J. (2004). Pure Appl. Chem..

[cit14] Merino E., Ribagorda M. (2012). Beilstein J. Org. Chem..

[cit15] Nicoletta F. P., Cupelli D., Formoso P., de Filpo G., Colella V., Gugliuzza A. (2012). Membranes.

[cit16] Banghart M., Borges K., Isacoff E., Trauner D., Kramer R. H. (2004). Nat. Neurosci..

[cit17] Houben S. J. A., Van Merwijk S. A., Langers B. J. H., Oosterlaken B. M., Borneman Z., Schenning A. P. H. J. (2021). ACS Appl. Mater. Interfaces.

[cit18] Hamaguchi K., Ichikawa R., Kajiyama S., Torii S., Hayashi Y., Kumaki J., Katayama H., Kato T. (2021). ACS Appl. Mater. Interfaces.

[cit19] Zhang Y., Dong R., Gabinet U. R., Poling-Skutvik R., Kim N. K., Lee C., Imran O. Q., Feng X., Osuji C. O. (2021). ACS Nano.

[cit20] Gracia I., Romero P., Serrano J. L., Barberá J., Omenat A. (2017). J. Mater. Chem. C.

[cit21] Li C., Cho J., Yamada K., Hashizume D., Araoka F., Takezoe H., Aida T., Ishida Y. (2015). Nat. Commun..

[cit22] Kishikawa K., Hirai A., Kohmoto S. (2008). Chem. Mater..

[cit23] Lee H. K., Lee H., Ko Y. H., Chang Y. J., Oh N. K., Zin W. C., Kim O. (2001). Angew. Chem., Int. Ed..

[cit24] Gin D. L., Lu X., Nemade P. R., Pecinovsky C. S., Xu Y., Zhou M. (2006). Adv. Funct. Mater..

[cit25] Hatakeyama E. S., Wiesenauer B. R., Gabriel C. J., Noble R. D., Gin D. L. (2010). Chem. Mater..

[cit26] Carter B. M., Wiesenauer B. R., Hatakeyama E. S., Barton J. L., Noble R. D., Gin D. L. (2012). Chem. Mater..

[cit27] Gupta M., Suzuki Y., Sakamoto T., Yoshio M., Torii S., Katayama H., Kato T. (2019). ACS Macro Lett..

[cit28] Sakamoto T., Ogawa T., Nada H., Nakatsuji K., Mitani M., Soberats B., Kawata K., Yoshio M., Tomioka H., Sasaki T., Kimura M., Henmi M., Kato T. (2018). Adv. Sci..

[cit29] Marets N., Kuo D., Torrey J. R., Sakamoto T., Henmi M., Katayama H., Kato T. (2017). Adv. Healthcare Mater..

[cit30] Henmi M., Nakatsuji K., Ichikawa T., Tomioka H., Sakamoto T., Yoshio M., Kato T. (2012). Adv. Mater..

[cit31] Feng X., Kawabata K., Kaufman G., Elimelech M., Osuji C. O. (2017). ACS Nano.

[cit32] Van Kuringen H. P. C., Leijten Z. J. W. A., Gelebart A. H., Mulder D. J., Portale G., Broer D. J., Schenning A. P. H. J. (2015). Macromolecules.

[cit33] Lugger J. A. M., Román P. P. M. S., Kroonen C. C. E., Sijbesma R. P. (2021). ACS Appl. Mater. Interfaces.

[cit34] Zeng H., Liang T., Zhang H., Wang Y., Wen J., Yu H., Cheng C. (2022). New J. Chem..

[cit35] Shishmanova I. K., Bastiaansen C. W. M., Schenning A. P. H. J., Broer D. J. (2012). Chem. Commun..

[cit36] Van Kuringen H. P. C., Eikelboom G. M., Shishmanova I. K., Broer D. J., Schenning A. P. H. J. (2014). Adv. Funct. Mater..

[cit37] Kato T., Fréchet J. M. J. (2006). Liq. Cryst..

[cit38] Patil S. V., Athare S. V., Jagtap A., Kodam K. M., Gejji S. P., Malkhede D. D. (2016). RSC Adv..

