# Supplementary material for: Photo-responsive nanoporous liquid crystal polymer films for selective dye adsorption
Source: RSC Adv. 2024 Jan 2;14(2):863–71. doi: 10.1039/d3ra06791b (PMC10759169; doi:10.1039/d3ra06791b)
Supplement: RA-014-D3RA06791B-s001 [file RA-014-D3RA06791B-s001.pdf]

Electronic Supplementary Material (ESI) for RSC Advances.
This journal is © The Royal Society of Chemistry 2023

Supporting Information

Photo-responsive nanoporous liquid crystal polymer film for selective dye adsorption

Hongju Zeng[1,3], Yun Wang[1], Changxiang Li[1], Junjie Ren[1], Runzi Lu[1], Huiyao Zhang[1], Xi Wang[1], Xingbin Lv[1,2], Hairong Yu[1,2], Ting Liang[*1,2] and Changjing Cheng[*1,2]

1. College of Chemistry and Environment, Southwest Minzu University, Chengdu, Sichuan 610200, PR China.

2. Key Laboratory of Fundamental Chemistry of the State Ethnic Commission, Chengdu, Sichuan 610200, PR China.

3. Chengdu BOE Display Sci-tech Co. Ltd., Chengdu, Sichuan 610200, PR China.

Email: liangting@swun.edu.cn; chengcj@swun.edu.cn

ORCID identifiers:

Hai-Rong Yu: 0000-0003-4925-2666

Xing-Bin Lv: 0009-0004-5582-221X

Ting Liang: 0000-0002-5824-9587

Chang-Jing Cheng: 0000-0002-2065-7240

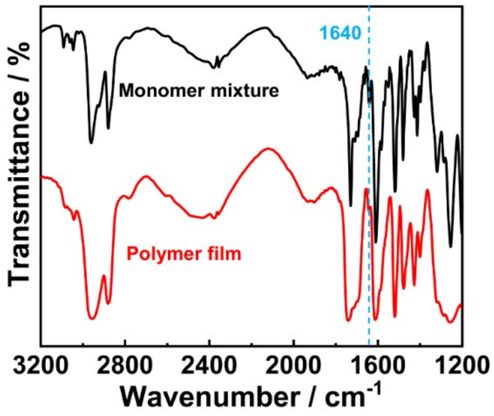

Figure S1. FT-IR spectra of the monomer mixture and the polymer film.

Table S1. Kinetic parameters of Rh6G adsorption into the **AZO-R (NC6)** film.

C_0 (mg/L)	$q_{e,exp}$ (mg/g)	Pseudo-first-order			Pseudo-second-order		
		$q_{e,cal}$ (mg/g)	R^2	k_1 (min^{-1})	$q_{e,cal}$ (mg/g)	R^2	k_2 (g/(mg·min))
5	13.74	7.35	0.9607	0.0393	14.37	0.9998	0.0113

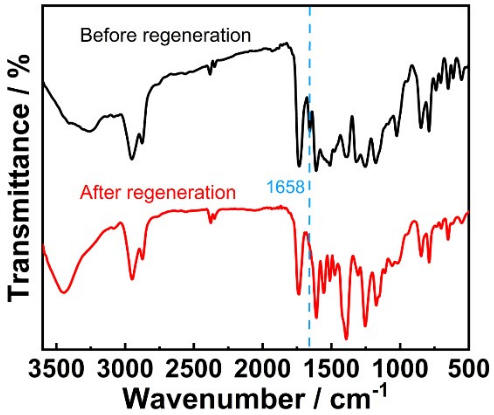

Figure S2. FT-IR spectra before and after regeneration.
